# Levels of Influenza A Virus Defective Viral Genomes Determine Pathogenesis in the BALB/c Mouse Model

**DOI:** 10.1128/jvi.01178-22

**Published:** 2022-10-13

**Authors:** Rebecca Penn, John S. Tregoning, Katie E. Flight, Laury Baillon, Rebecca Frise, Daniel H. Goldhill, Cecilia Johansson, Wendy S. Barclay

**Affiliations:** a Department of Infectious Disease, Imperial College London, London, United Kingdom; b National Heart and Lung Institute, Imperial College London, London, United Kingdom; Lerner Research Institute, Cleveland Clinic

**Keywords:** defective viral genomes, influenza, pathogenesis

## Abstract

Defective viral genomes (DVGs), which are generated by the viral polymerase in error during RNA replication, can trigger innate immunity and are implicated in altering the clinical outcome of infection. Here, we investigated the impact of DVGs on innate immunity and pathogenicity in a BALB/c mouse model of influenza virus infection. We generated stocks of influenza viruses containing the internal genes of an H5N1 virus that contained different levels of DVGs (indicated by different genome-to-PFU ratios). In lung epithelial cells, the high-DVG stock was immunostimulatory at early time points postinfection. DVGs were amplified during virus replication in myeloid immune cells and triggered proinflammatory cytokine production. In the mouse model, infection with the different virus stocks produced divergent outcomes. The high-DVG stock induced an early type I interferon (IFN) response that limited viral replication in the lungs, resulting in minimal weight loss. In contrast, the virus stock with low levels of DVGs replicated to high titers and amplified DVGs over time, resulting in elevated levels of proinflammatory cytokines accompanied by rapid weight loss and increased morbidity and mortality. Our results suggest that the timing and levels of immunostimulatory DVGs generated during infection contribute to H5N1 pathogenesis.

**IMPORTANCE** Mammalian infections with highly pathogenic avian influenza viruses (HPAIVs) cause severe disease associated with excessive proinflammatory cytokine production. Aberrant replication products, such as defective viral genomes (DVGs), can stimulate the antiviral response, and cytokine induction is associated with their emergence *in vivo*. We show that stocks of a recombinant virus containing HPAIV internal genes that differ in their amounts of DVGs have vastly diverse outcomes in a mouse model. The high-DVG stock resulted in extremely mild disease due to suppression of viral replication. Conversely, the stock that contained low DVGs but rapidly accumulated DVGs over the course of infection led to severe disease. Therefore, the timing of DVG amplification and proinflammatory cytokine production impact disease outcome, and these findings demonstrate that not all DVG generation reduces viral virulence. This study also emphasizes the crucial requirement to examine the quality of virus preparations regarding DVG content to ensure reproducible research.

## INTRODUCTION

Zoonotic infections of highly pathogenic avian influenza virus (HPAIV), such as the H5N1 subtype, pose a significant threat to public health. HPAIV infections in mammalian hosts are associated with a dysregulated innate immune response known as a cytokine storm (hypercytokinemia). This leads to increased damage to host tissues and acute respiratory distress syndrome (ARDS), resulting in high fatality rates (1–4). The host and viral factors leading to the HPAIV mammalian cytokine storm are still poorly defined, and attempts to further our understanding could be pivotal in guiding therapeutic options in future outbreaks.

Detection of pathogens by the innate immune system is mediated through pattern recognition receptors (PRRs), which are expressed in host cells and specifically recognize and bind pathogen-associated molecular patterns (PAMPs). One such PAMP is virally derived nucleic acids. Once bound, signaling cascades induce the production of both cytokines and chemokines, including the type I interferons. Type I IFN signaling via the interferon alpha/beta (IFN-α/β) receptor (IFNAR) culminates in the expression of numerous IFN-stimulated genes (ISGs), many of which encode proteins that have antiviral functions, thus serving to curb viral replication and minimize viral spread (5). While this is often beneficial for the host, if type I IFN is produced in excess or mistimed, it can lead to detrimental consequences, particularly through inflammation (6, 7). Indeed, increased type I IFN production has been linked to elevated host inflammatory responses and, alongside high levels of interleukin-6 (IL-6), tumor necrosis factor (TNF), IL-8, interferon gamma-induced protein 10 (IP-10) (CXCL10), and monocyte chemoattractant protein 1 (MCP-1) (CCL2), is a hallmark of H5N1 HPAIV infection in mammals (8–10).

During viral replication, aberrant replication products can be generated, and these have also been shown to act as PAMPs, as they share common features with the full-length (FL) genome, such as a short length of double-stranded RNA and a 5′ terminal triphosphate moiety, both of which are recognized by the PRR RIG-I (11, 12). The most well-known viral aberrant RNA species are defective viral genomes (DVGs), which were historically thought to be artifacts created by passaging viruses at high multiplicities of infection (MOIs) *in vitro* (13). However, numerous studies have demonstrated the presence of DVGs generated by natural infections with a wide range of RNA viruses (14–17). Different types of DVGs have been characterized, but most lack large parts of the viral genome; deletion DVGs retain the 5′ and 3′ termini and RNA structural elements necessary for replication, but lack the RNA core (18), whereas copy-back DVGs (cbDVGs) lack the central portion of RNA but contain the 5′ end with a mutated and perfectly complementary 3′ end forming a panhandle structure (19). More recently, another type of influenza virus deletion DVG, termed a mini-viral RNA (mvRNA) was discovered; these are small DVGs, less than 125 nucleotides (nt) in length (20).

As many DVGs retain core packaging signals, they can be packaged into newly formed virions, but they are noninfectious since they will not be replicated unless a fully infectious particle or a particle containing the complementary full-length segment also coinfects the same cell. These noninfectious particles are often referred to as defective interfering particles (DIPs), as they interfere with standard viral replication by competing with full-length segments for polymerase components and packaging and can reduce overall virus titers *in vitro* (21). This feature, coupled with their ability to trigger type I IFN, has led to numerous studies investigating their applicability as broad-spectrum antivirals (22–25). It has also been shown that, in animal models, the administration of influenza DIPs prior to influenza virus challenge is protective and increases survival rates (26–28). Similarly, higher levels of DVGs present in virus stocks used to conduct animal studies led to milder disease and high levels of DVGs, which correlates with milder infections in humans (29, 30). However, the role of DVGs in the cytokine storm seen when HPAIVs infect mammalian hosts is still unclear. Previous work has shown that more mvRNAs were generated in human cells by the H5N1 HPAIV polymerase than by a mammalian-adapted influenza polymerase. Moreover, high levels of mvRNAs were detected in the lungs of ferrets infected with either an HPAIV H5N1 or the 1918 pandemic H1N1 strain and correlated with increased proinflammatory cytokine expression and cell death (20). Interestingly, a novel protein encoded by a DVG derived from the polymerase basic 2 protein (PB2) segment of an H5N1 virus strain has also been shown to induce type I IFN and lead to enhanced disease severity in a mouse model (31).

In this study, we addressed whether DVGs contribute to pathogenesis following infection with a virus containing the internal genes of an HPAIV H5N1 virus in BALB/c mice. Previous work in our laboratory has shown that viruses containing the internal genes of HPAIV H5N1 cause dramatic weight loss that is driven by high cytokine production rather than high viral lung load (32). We wished to ascertain whether DVGs would exacerbate the hyperactivation of the innate immune response *in vivo.* We hypothesized that DVGs amplified during infection would drive hypercytokinemia and immunopathology in the lung, resulting in a more severe infection. We therefore generated stocks of influenza viruses containing the internal genes of an H5N1 HPAIV that varied in their DVG levels. We analyzed the accumulation of both full-length genomes and DVGs *in vitro* and *in vivo*, as well as infectious viral loads and the induction of type I IFNs and other proinflammatory cytokines and chemokines. Our studies reveal that the amount of DVGs in the initial virus inoculum, as well as their sustained replication, can have a profound impact on the severity of the infection.

## RESULTS

### Virus stocks grown at different MOIs contain various levels of DVGs.

**FIG 1:**
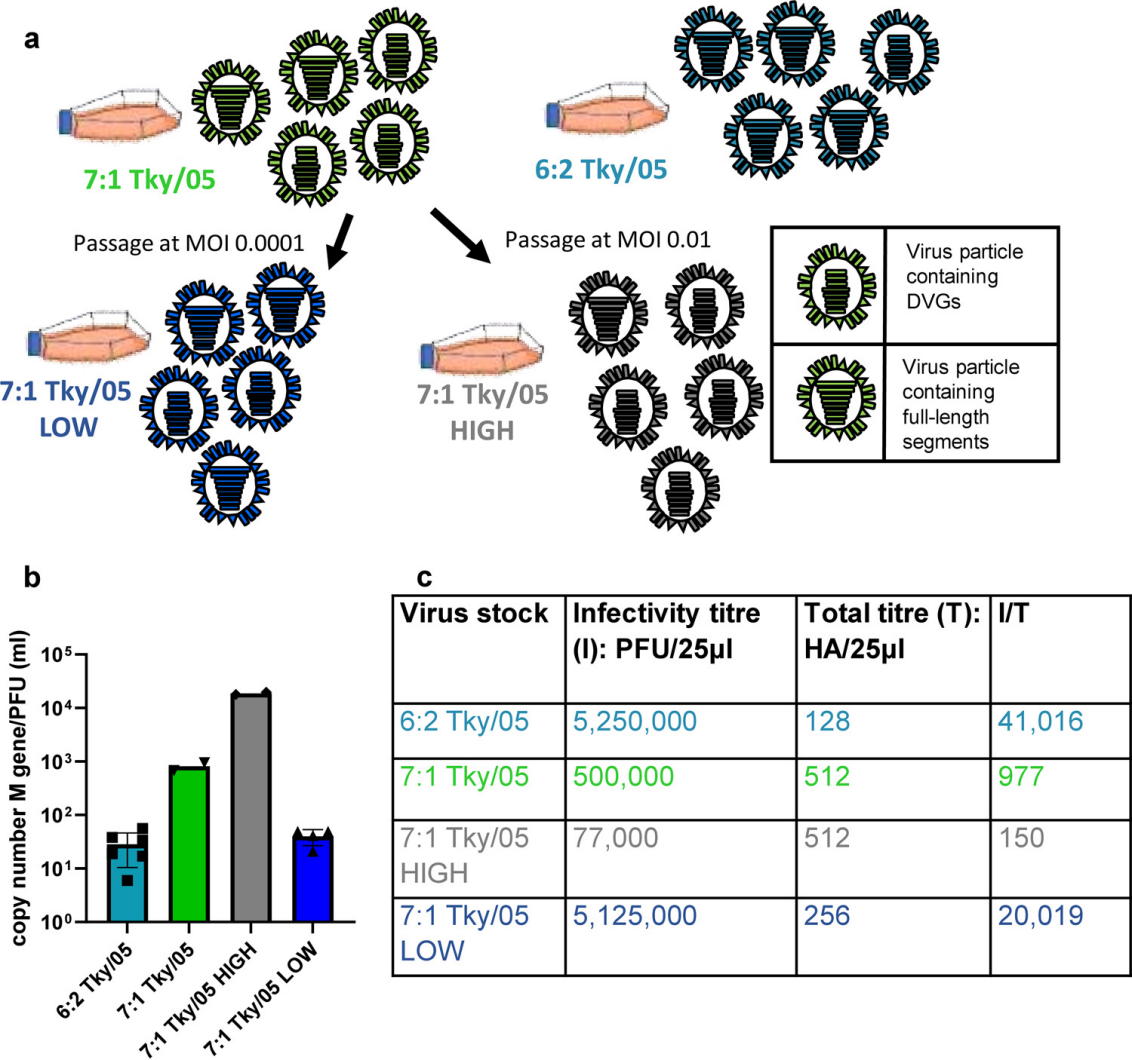
Virus stocks grown at different MOIs contain different amounts of infectious viral particles. (a) Schematic showing the generation of the virus stocks used in this study. (b) Ratio of genome copy number/mL to PFU/mL. Mean PFU/mL was determined for each virus stock (*n* = 3), and this value was used to calculate the ratio. RNA was extracted from viral stocks, and a one-step RT-qPCR performed using primers and probe for the M gene to calculate the M gene copy number/mL. Data points show ratios calculated from RT-qPCR using RNA obtained from at least two independent extractions. Bars and error bars represent mean values ± SD. (c) Infectious-to-total (*I*/*T*) particle ratios in virus stocks.

To grow stocks of virus with differing levels of DVGs, we used a virus rescued using plasmids bearing the six internal genes and the neuraminidase (NA) gene from the HPAIV H5N1 virus A/turkey/Turkey/1/2005 (Tky/05) and the hemagglutinin (HA) from the H1N1 laboratory strain A/Puerto Rico/8/34 (PR8). This stock (7:1 Tky/05 virus) had been passaged multiple times with different MOIs during laboratory propagation and was experimentally determined to contain high levels of DVGs. The 7:1 Tky/05 virus stock was then further passaged in MDCK cells at two different MOIs; an MOI of 0.01 generated 7:1 Tky/05 HIGH, and an MOI of 0.0001 generated 7:1 Tky/05 LOW (Fig. 1a). We also rescued an additional virus that contained the same internal genes but had genes for both HA and NA derived from PR8. This 6:2 Tky/05 virus was passaged in MDCK cells at a low MOI (0.0001) directly from the original viral rescue to minimize the generation of DVGs. The matrix protein (M) gene copy number-to-PFU ratios were similar for both viruses passaged at low MOIs, with ratios of 28:1 for 6:2 Tky/05 and 40:1 for 7:1 Tky/05 LOW, but the ratio was substantially higher for the 7:1 Tky/05, at 813:1, and grossly elevated for the 7:1 Tky/05 HIGH stock, at 18,781:1 (Fig. 1b). We also calculated the infectious titer (*I*)/total particle (*T*) ratios; these were lower for both the 7:1 Tky/05 and the 7:1 Tky/05 HIGH virus, indicating that the proportions of particles that were infectious were lower in these virus preparations (Fig. 1c).

**FIG 2:**
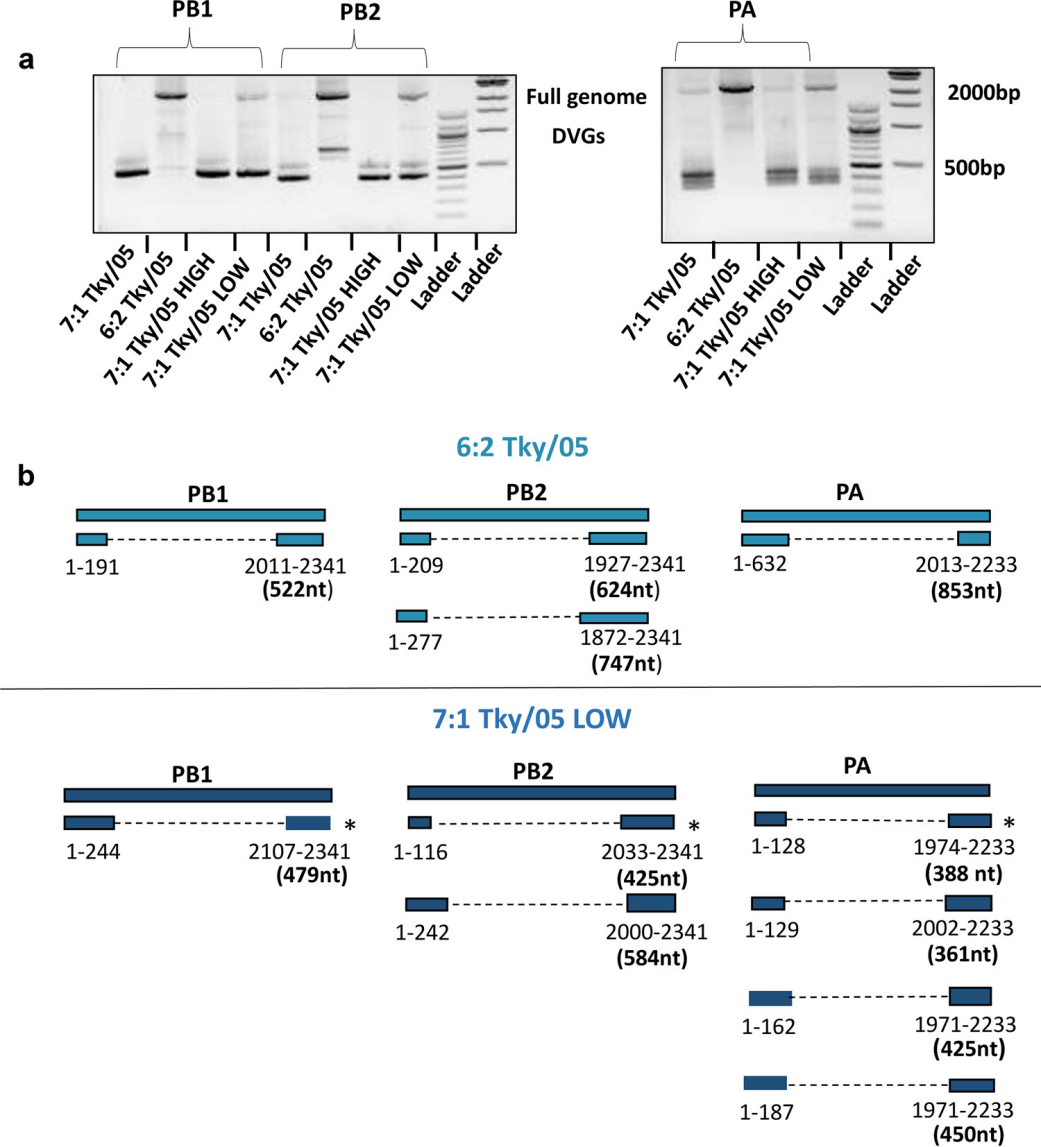
Characterization of DVGs identified in virus stocks. (a) Gel electrophoresis of RT-PCR products of DVGs and full genomes from viral stocks using segment-specific terminal primers for the polymerase genes. (b) DVGs identified in viral stocks through Sanger sequencing of TOPO-cloned RT-PCR products for PB1, PB2, and PA segments. All bands not corresponding to the full-length genomes (presumed to be DVGs) from the 6:2 Tky/05 and 7:1 Tky/05 LOW virus stocks were cloned and sequenced. One PCR product from each polymerase segment (band sizes are 479 nt for PB1, 425 nt for PB2, and 388 nt for PA) was also cloned and sequenced from the 7:1 Tky/05 and 7:1 Tky/05 HIGH virus stocks, and these were identical in sequence to those present in the 7:1 Tky/05 LOW stock (marked by asterisks).

To characterize the DVG content in these stocks, we employed reverse transcription (RT)-PCR using primers that bind to the 3′ and 5′ ends of the polymerase segments [polymerase basic 1 (PB1), polymerase basic 2 (PB2) and polymerase acidic (PA)] to amplify full-length viral genomes and DVGs. We focused on the polymerase genes because a higher abundance of DVGs are typically derived from these segments (33). Indeed, small products (361 to 853 nt) derived from all three polymerase segments were detected in all virus stocks, although at much higher levels in the 7:1 Tky/05 and 7:1 Tky/05 HIGH stocks and, interestingly, also in the 7:1 Tky/05 LOW stock (Fig. 2a). The poor amplification of the full-length segments for the 7:1 Tky/05 and 7:1 Tky/05 HIGH viruses provides further evidence that these stocks contain high levels of DVGs. Sanger sequencing of the short PCR products confirmed that they retained the termini but contained large internal deletions with different junctions (Fig. 2b). The virus populations originating from the 7:1 Tky/05 stocks (7:1 Tky/05 HIGH and 7:1 Tky/05 LOW) contained identical DVGs but differed in their abundance. DVGs identified from the independently rescued 6:2 Tky/05 virus were genetically distinct. Most of the DVGs identified were between 350 and 550 nt in length, which is the length previously found to be enriched both in an artificial DVG library and in natural infections (34, 35).

### Intracellular DVGs accumulate early postinfection and have strong immunostimulatory activity.

**FIG 3:**
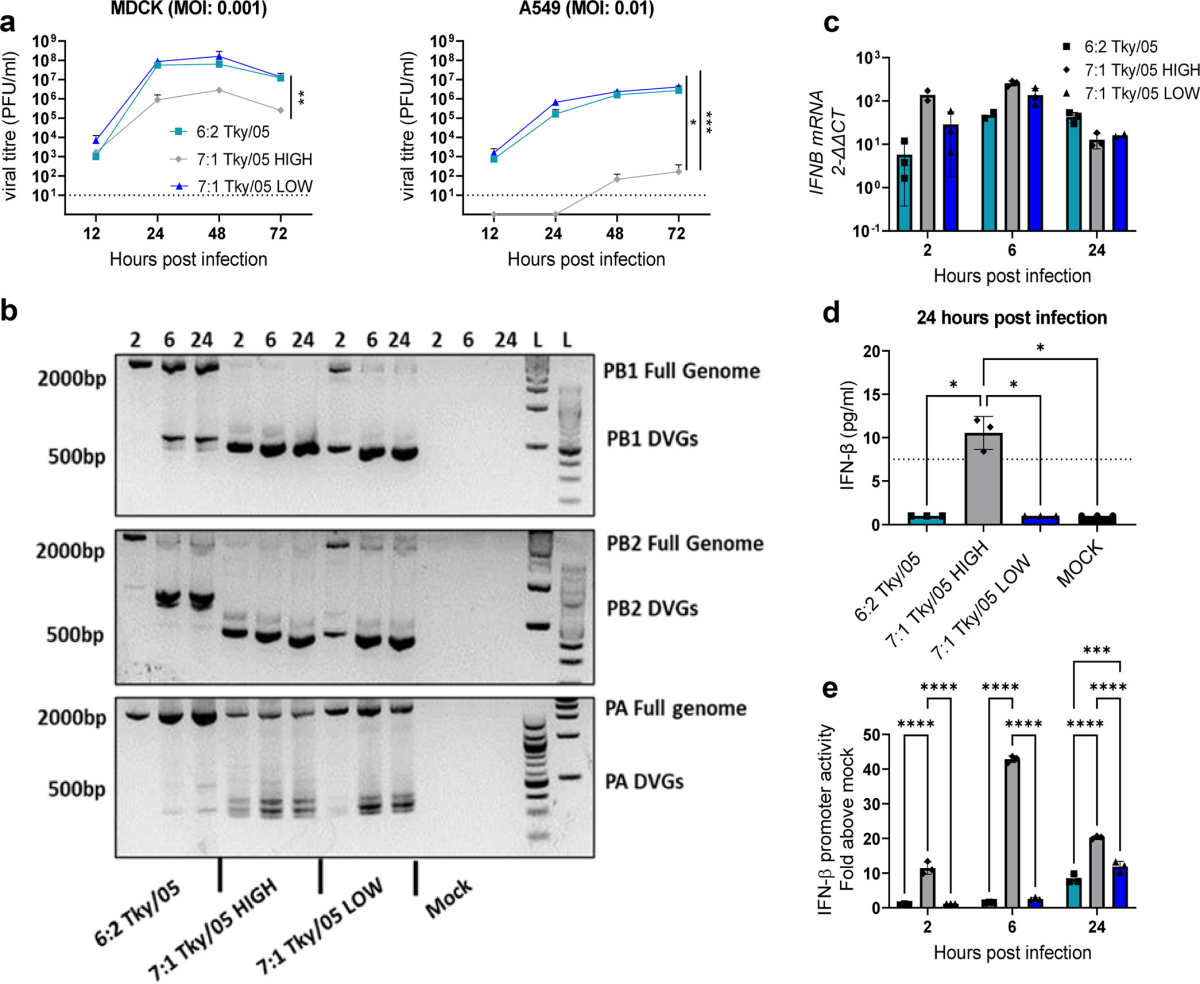
Growth kinetics, accumulation of DVGs, and type I IFN induction in infected cells. (a) Growth kinetics of 6:2 Tky/05, 7:1 Tky/05 HIGH, and 7:1 Tky/05 LOW viruses in MDCK and A549 cells. MOIs used for infections are displayed and were normalized by PFU. At designated time points, supernatants were taken and plaque assays performed to determine viral titers. Data are shown as mean values ± standard deviations (SD) (*n* = 3). Dotted lines represent the limit of detection. (b) A549 cells were infected using the viruses whose growth kinetics are shown in panel a at an MOI of 1 or mock infected. At 2, 6, and 24 h.p.i., total RNA was extracted, and RT-PCR was performed using terminal primers to detect full genomes and DVGs (*n* = 3). The gels each show the results for one representative well. (c) A549 cells were infected using the viruses whose growth kinetics are shown in panel a at an MOI of 1 or mock infected. At 2, 6, and 24 h.p.i., total RNA was extracted, and RT-qPCR was performed using primers for *IFNB*. Relative expression was calculated by the 2^−ΔΔ^*^CT^* method and normalized to the results for the *GAPDH* housekeeping gene, and data points represent the fold increase over the value for the mock-infected cells. Bars and error bars represent the mean values from 2 or 3 biological replicates ± SD. (d) IFN-β protein levels in A549 cell supernatants at 24 h.p.i. as determined by ELISA. Bars and error bars represent mean values ± SD (*n* = 3). Dotted line represents the limit of detection. (e) A549 IFN-β luciferase reporter cells were infected with an MOI of 1 of the same virus stocks whose growth kinetics are shown in panel a or mock infected, and at 2, 6, and 24 h.p.i., cells were lysed and luciferase expression measured. Bars and error bars represent mean values ± SD (*n* = 3). Variance among groups was calculated by two-way analysis of variance (ANOVA) with Tukey’s *post hoc* test for multiple comparisons. *, *P* < 0.05; ***, *P* < 0.001; ****, *P* < 0.0001.

To investigate how growth kinetics *in vitro* were affected by DVG content, the 6:2 Tky/05, 7:1 Tky/05 HIGH, and 7:1 Tky/05 LOW viruses were used to infect MDCK cells or human alveolar lung epithelial cells (A549). The viruses released into supernatants were quantified over a 72-h time period (Fig. 3a). The 6:2 Tky/05 and 7:1 Tky/05 LOW viruses showed similar replication kinetics, whereas the 7:1 Tky/05 HIGH stock yielded significantly lower infectious titers in both cell types at 24, 48, and 72 h postinfection (h.p.i.).

We tracked the amplification of DVGs in cells during infection. A549 cells were infected at an MOI of 1 (based on PFU) with the 6:2 Tky/05, 7:1 Tky/05 HIGH, or 7:1 Tky/05 LOW virus stock, and total RNA was harvested at 2, 6, and 24 h. RT-PCR analysis showed the presence of DVGs with band sizes correlating with those present in the original viral stocks (Fig. 3b). Abundant DVGs were detected at 2 h after infection with the 7:1 Tky/05 HIGH, likely representing incoming DVGs from the virus inoculum. All the infections resulted in intracellular DVGs that accumulated over time, regardless of the virus stock used for infection. As virus stocks containing high levels of DVGs induce type I IFN (36–39), we next assessed whether the 7:1 Tky/05 HIGH stock triggered higher levels of type I IFNs and whether there was an impact on the kinetics of IFN expression. *IFNB* mRNA expression was determined by RT-quantitative PCR (RT-qPCR) using total RNA from the same experiment. In addition, we detected secreted IFN-β protein in the supernatants by enzyme-linked immunosorbent assay (ELISA). At both 2 and 6 h.p.i., the *IFNB* mRNA levels were highest for the 7:1 Tky/05 HIGH virus (Fig. 3c). IFN-β protein was only detected in the supernatants harvested from cells infected with this virus (Fig. 3d). We next used an A549 IFN-β luciferase (Luc) reporter cell line to measure IFN-β promoter activity at 2, 6, and 24 h.p.i. (Fig. 3e). In line with the previous results, only the 7:1 Tky/05 HIGH stock induced a detectable increase of IFN-β promoter activity over the level in mock-infected cells at 2 h.p.i., peaking at 6 h. Therefore, IFN-β induction in infected human lung cells reflected DVG accumulation rather than infectious virus, agreeing with previous findings (36, 38).

### Intracellular DVGs accumulate in macrophages and trigger proinflammatory cytokines.

**FIG 4:**
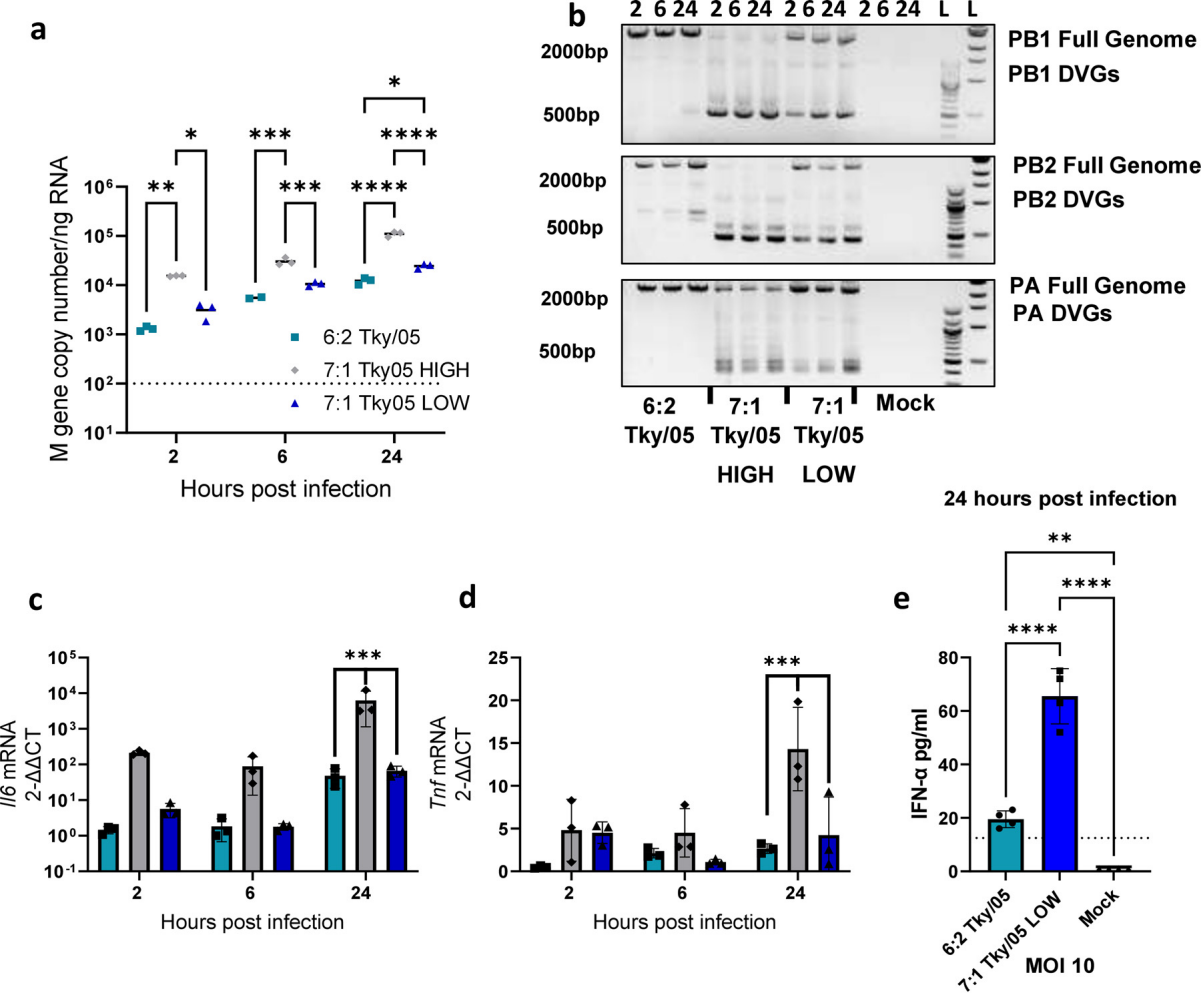
DVGs replicate in BMDMs and trigger proinflammatory cytokines. (a) BMDMs were infected at an MOI of 1 with the 6:2 Tky/05, 7:1 Tky/05 HIGH, and 7:1 Tky/05 LOW virus stocks or mock infected (*n* = 3). At 2, 6, and 24 h.p.i., total RNA was extracted, and one-step RT-qPCR was performed using primers and probe for the M gene. Horizontal bars represent the mean values. (b) Gel electrophoresis of RT-PCR products using segment-specific terminal primers to detect full-length polymerase genes and DVGs from infected BMDMs. The gels each show the results for one representative well. (c and d) RT-qPCR for mRNA *Il6* and *Tnf* gene expression was performed on infected BMDMs. Relative expression was calculated by the 2^−ΔΔ^*^CT^* method and normalized to the value for the *Gapdh* housekeeping gene, and data points represent the fold increase over the value for the mock-infected cells. Bars and error bars represent the mean values ± SD (*n* = 3). Variance among groups was calculated by two-way ANOVA with Tukey’s *post hoc* test for multiple comparisons. (e) BMDMs were infected at an MOI of 10 with the 6:2 Tky/05 and 7:1 Tky/05 LOW virus stocks or mock infected (*n* = 4). At 24 h.p.i., IFN-α protein levels in supernatants were determined by ELISA. Dotted line represents the limit of detection. Bars and error bars represent the mean values ± SD. Variance among the groups was determined by one-way ANOVA with Tukey’s *post hoc* test for multiple comparisons. *, *P* < 0.05; **, *P* < 0.01; ***, *P* < 0.001; ****, *P* < 0.0001.

Although epithelial cells are the main targets for influenza virus infection and are the source of progeny viral particles, myeloid immune cells, such as dendritic cells (DCs) and macrophages, can also be infected (40). Sensing viral RNA in these cell types leads to the production of cytokines; both macrophages and DCs have been shown to secrete high levels of proinflammatory cytokines following infection with HPAIV H5N1 and have been implicated in the pathogenesis of H5N1 mammalian infections (9, 32). We therefore wanted to address whether the viruses in our study replicated in macrophages and, more specifically, whether DVGs would be generated in these cells. We infected murine bone marrow-derived macrophages (BMDMs) with an MOI of 1 of the 6:2 Tky/05, 7:1 Tky/05 HIGH, or 7:1 Tky/05 LOW virus stocks and harvested total RNA at 2, 6, and 24 h. Copy numbers of the M gene increased over the 24-h time period for all viruses, indicating that the viruses were replicating within these cells (Fig. 4a). At 2 h.p.i., the 7:1 Tky/05 HIGH virus M gene copy number was significantly higher than that of either the 6:2 Tky/05 or the 7:1 Tky/05 LOW virus, which is reflective of the higher number of genomes in the starting inoculum, as the input was normalized by PFU. As seen in epithelial cells, DVGs were readily detected in macrophages when the polymerase segments were analyzed by RT-PCR, and most bands were of similar sizes to those present in the original virus stocks (Fig. 4b). *Il6* (Fig. 4c) and *Tnf* (Fig. 4d) mRNAs were significantly upregulated at 24 h.p.i. following infection with the 7:1 Tky/05 HIGH virus. There was no difference observed between the 6:2 Tky/05 and 7:1 Tky/05 LOW viruses. However, a significant difference in the amounts of secreted IFN-α at 24 h.p.i. was observed when the 6:2 Tky/05 and 7:1 Tky/05 LOW viruses were used to infect the BMDMs at the higher MOI of 10. In this case, the 7:1 Tky/05 LOW virus resulted in significantly higher IFN-α production (Fig. 4e). Overall, this finding suggested that DVGs can induce the release of high levels of type I IFNs and proinflammatory cytokines from macrophages.

### The amount of DVGs in the inoculum impacts the infection outcome *in vivo*.

**FIG 5:**
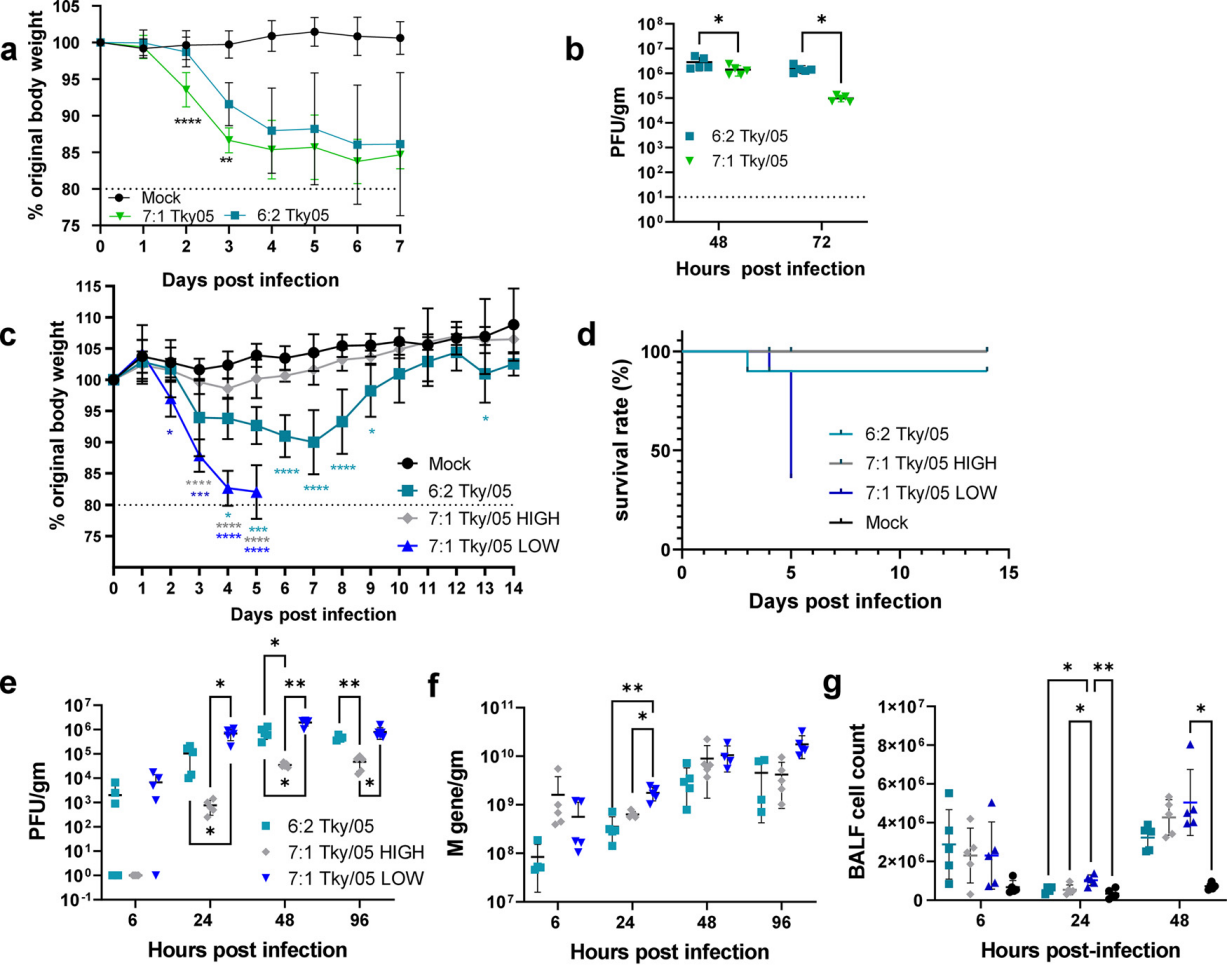
Tky/05 virus stocks containing different levels of DVGs have diverse infection outcomes. (a) Six- to 8-week-old female BALB/c mice (15 per group) were mock infected or infected intranasally with 10^5^ PFU of 6:2 Tky/05 and 7:1 Tky/05 virus in 25-μL volumes. Five mice per group were culled at 48 h, 72 h, and 7 days postinfection. Weight loss was monitored daily. Data represent the mean values ± SD. Differences between the 6:2 Tky/05- and 7:1 Tky/05 virus-infected mice were analyzed by two-way ANOVA with Tukey’s *post hoc* test for multiple comparisons. (b) Virus titers in homogenized lung tissues were determined by plaque assay at 48 and 72 h postinfection. Horizontal bars represent the mean values. (c) Six- to 8-week-old female BALB/c mice (25 per group) were mock infected (PBS) or infected intranasally with 10^5^ PFU of 6:2 Tky/05, 7:1 Tky/05 HIGH, or 7:1 Tky/05 LOW virus in 35-μL volumes. Five mice per group were culled at 6 h, 24 h, 48 h, 96 h, and 14 days postinfection. Weight loss was monitored daily. Data represent the mean values ± SD. Dark blue asterisks indicate statistically significant differences between mice infected with 6:2 Tky/05 and 7:1 Tky/05 LOW viruses, gray asterisks indicate statistically significant differences between mice infected with 7:1 Tky/05 HIGH and 7:1 Tky/05 LOW viruses, and teal asterisks indicate statistically significant differences between mice infected with 7:1 Tky/05 HIGH and 6:2 Tky/05 viruses. Dotted line represents the severity limit. (d) Survival curves of infected mice. All mice were culled when they lost ≥20% of their original body weight (day zero). (e) Virus titers in homogenized lung tissues were determined by plaque assay at 6, 24, 48, and 96 h.p.i. (*n* = 5 per group). Horizontal bars and error bars represent the mean values ± SD. (f) RNA was extracted from the homogenized lungs, and one-step RT-qPCR performed using primers and probe for the M gene (*n* = 5 per group). Horizontal bars and error bars represent mean values ± SD. (g) BAL fluid was obtained at 6, 24, and 48 h.p.i., and cell counts calculated (*n* = 5 per group). Horizontal bars and error bars represent the mean values ± SD. Variance among groups was calculated by two-way ANOVA with Tukey’s *post hoc* test for multiple comparisons. *, *P* < 0.05; **, *P* < 0.01; ***, *P* < 0.001; ****, *P* < 0.0001.

To assess the impact of DVGs on pathogenicity, 6- to 8-week-old BALB/c mice were intranasally infected with 10^5^ PFU of the virus stocks. In an initial mouse experiment, the 6:2 Tky/05 and 7:1 Tky/05 stocks were assessed. Both viruses induced weight loss compared to the body weight of the mock-infected mice, but the group infected with 7:1 Tky/05 virus lost weight at a higher rate (Fig. 5a). Viral lung loads were measured by plaque assay, and at both time points measured, lower levels of infectious virus were recovered from the 7:1 Tky/05-infected murine lungs (Fig. 5b). To perform a detailed exploration of the link between cytokines, viral load, and disease, we infected mice with equivalent doses of the 6:2 Tky/05, 7:1 Tky/05 HIGH, and 7:1 Tky/05 LOW virus stocks. The mice infected with 6:2 Tky/05 and 7:1 Tky/05 LOW viruses lost significantly more weight than the healthy mock-infected control group (Fig. 5c). More rapid weight loss was observed for the 7:1 Tky/05 LOW-infected group; by day 5, all but one mouse had reached the 20% severity limit and all mice were culled (Fig. 5d). This is in direct contrast to the mice infected with the 7:1 Tky/05 HIGH virus, where there was no significant difference in weight loss over the 14-day time period compared to the weight in the mock-infected group. Lung homogenate viral loads measured by PFU followed a similar pattern to that observed *in vitro*, with significantly lower numbers of PFU/g in lungs from mice infected with 7:1 Tky/05 HIGH virus and higher numbers of PFU/g in the 7:1 Tky/05 LOW-infected lungs at all time points (Fig. 5e). At 24 h.p.i., the viral load as determined by M gene copy number/g was significantly higher for the 7:1 Tky/05 LOW-infected group (Fig. 5f). Cellular infiltration into the bronchoalveolar lavage (BAL) fluid was also higher at 24 h.p.i. in the 7:1 Tky/05 LOW-infected group (Fig. 5g).

**FIG 6:**
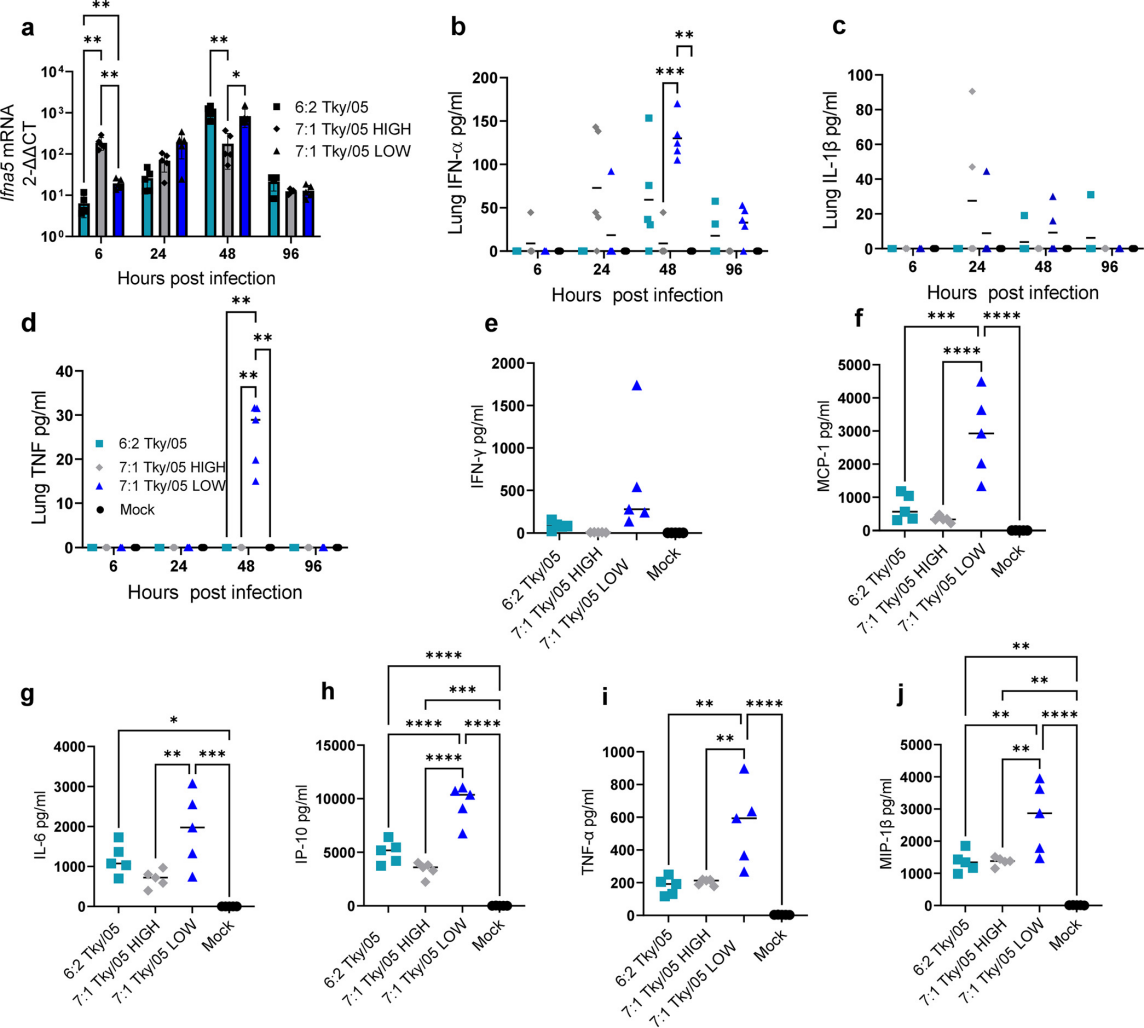
Levels of DVGs in the Tky/05 virus stocks impact cytokine production. (a) Total RNA was extracted from murine lungs, and RT-qPCR for *Ifna*5 gene expression was performed. Relative expression was calculated by the 2^−ΔΔ^*^CT^* method and normalized to the value for the *Gapdh* housekeeping gene, and data points show the fold increase over the value for the mock-infected cells. Bars and error bars represent mean values ± SD (*n* = 5). Variance among groups was calculated by two-way ANOVA with Tukey’s *post hoc* test for multiple comparisons. (b to d) IFN-α, TNF, and IL-1B protein levels in murine lung homogenates at 6, 24, 48, and 96 h.p.i. as determined by ELISA. Horizontal bars represent the mean values (*n* = 5). (e to j) IFN-γ, MCP-1, IL-6, IP-10, TNF, and MIP-1β protein levels in the BAL fluid at 48 h.p.i. Horizontal bars represents the mean values (*n* = 5). Variance among groups was calculated by one-way ANOVA with Tukey’s *post hoc* test for multiple comparisons. *, *P* < 0.05; **, *P* < 0.01; ***, *P* < 0.001; ****, *P* < 0.0001.

*Ifna5* mRNA peaked at 6 h.p.i. following infection with the 7:1 Tky/05 HIGH virus (Fig. 6a). In contrast, *Ifna5* mRNA expression peaked at 48 h following infection with either 6:2 Tky/05 or 7:1 Tky/05 LOW virus. This difference in the kinetics of IFN-α expression driven by the viruses was also observed for the protein levels in the murine lungs (Fig. 6b), with concurrent increases in IL-1β (Fig. 6c) and TNF (Fig. 6d). There were significantly higher levels of the proinflammatory cytokines IFN-γ (Fig. 6e), MCP-1 (Fig. 6f), IL-6 (Fig. 6g), IP-10 (Fig. 6h), TNF (Fig. 6i), and MIP-1β (Fig. 6j) than in the 6:2 Tky/05- and 7:1 Tky/05 HIGH-infected mice.

### DVGs are detected *in vivo*.

**FIG 7:**
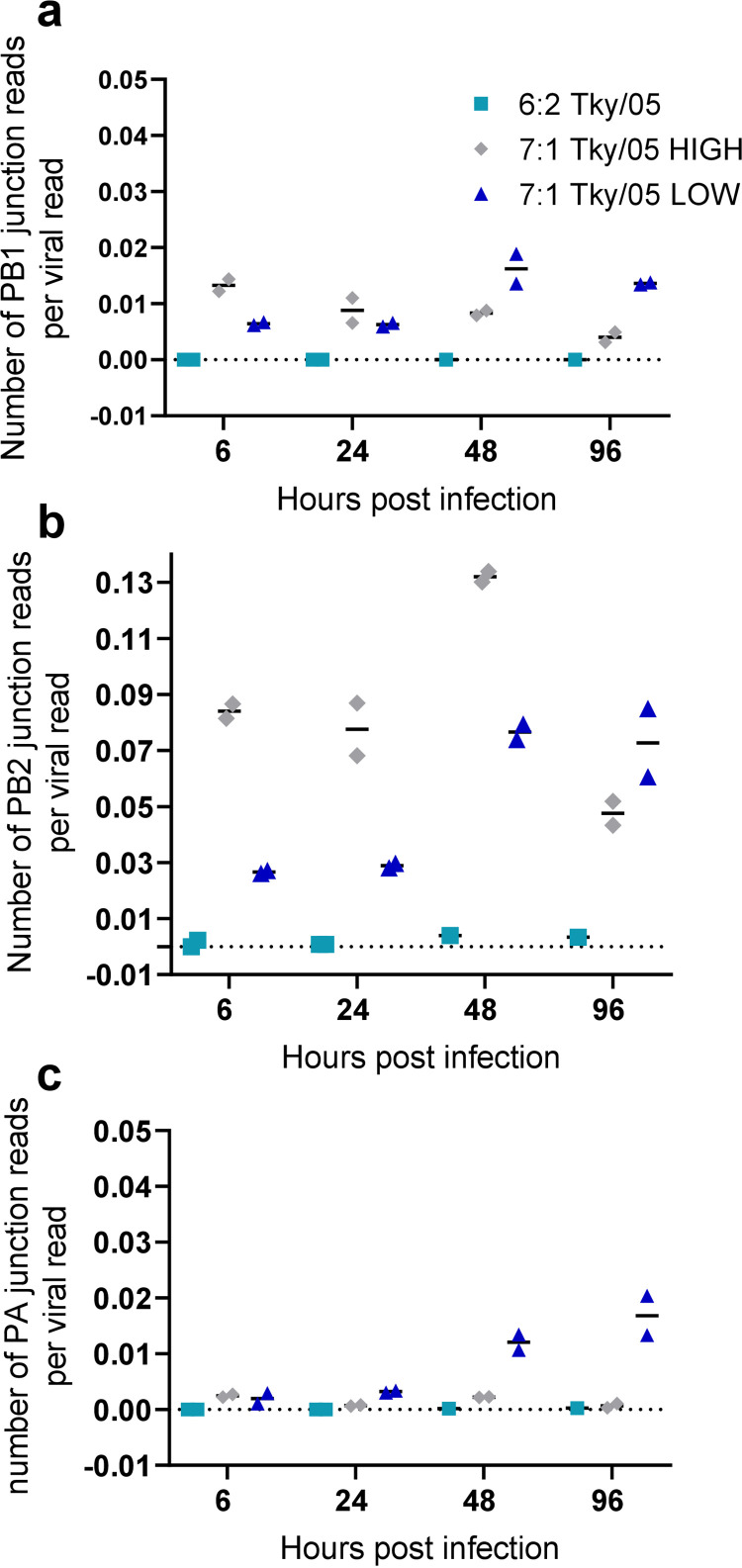
Number of DVG junction reads per viral read in infected murine lungs. RNA was extracted from the lungs of the infected mice, sequenced, and analyzed by ViReMa. The junction reads for PB1 (a), PB2 (b), and PA (c) were divided by the total number of viral reads per sample. Each data point represents the value for 1 mouse. Horizontal bars represent the mean values.

We characterised DVGs arising over the course of the infection by sequencing murine lungs using next-generation sequencing (NGS) and ViReMa (41) which has previously been shown to accurately characterise influenza DVGs (42). Analysis of two mice per time point (6, 24, 48, and 96 h.p.i.) for each virus was performed, apart from the 6:2 Tky/05 virus-infected cohort, where only 1 mouse was analyzed at 48 and 96 h.p.i., due to insufficient yield during NGS library construction. To compare DVG levels across the samples despite differences in the total numbers of viral reads present, we divided the total number of junction reads (detected by ViReMa) by the total number of mapped viral reads. Although this approach will not accurately quantify the frequencies of the defective viral genomes present in the viral population, due to uneven genome coverage and PCR bias for amplifying shorter-length products, it does allow relative comparisons between samples. At 6 h, we saw higher levels of all the polymerase DVGs (PB1 [Fig. 7a], PB2 [Fig. 7b], and [PA] [Fig. 7c]) for the mice infected with the 7:1 Tky/05 HIGH virus stock. However, by 96 h.p.i., there were higher numbers of DVGs detected in the 7:1 Tky/05 LOW virus-infected murine lungs. Interestingly, the highest levels of DVGs detected were those from the PB2 segment for all mice across all time points, and this segment generated the most diversity (Fig. 7 and Table S1 in the supplemental material). Many of the DVGs detected in the lungs were those previously cloned and Sanger sequenced from the viral stock (Fig. 2b), and these remained the most abundant DVGs at all time points. Very few DVGs mapped to nonpolymerase segments, except for a DVG generated from the nucleoprotein (NP) segment in the lungs of multiple mice infected with each of the virus stocks at various time points. We also observed an HA-derived DVG that was seen in one of the lungs harvested at 6 h.p.i. from the 7:1 Tky/05 HIGH-infected group (Table S1).

### DVGs cloned from the 7:1 Tky/05 virus are immunostimulatory.

**FIG 8:**
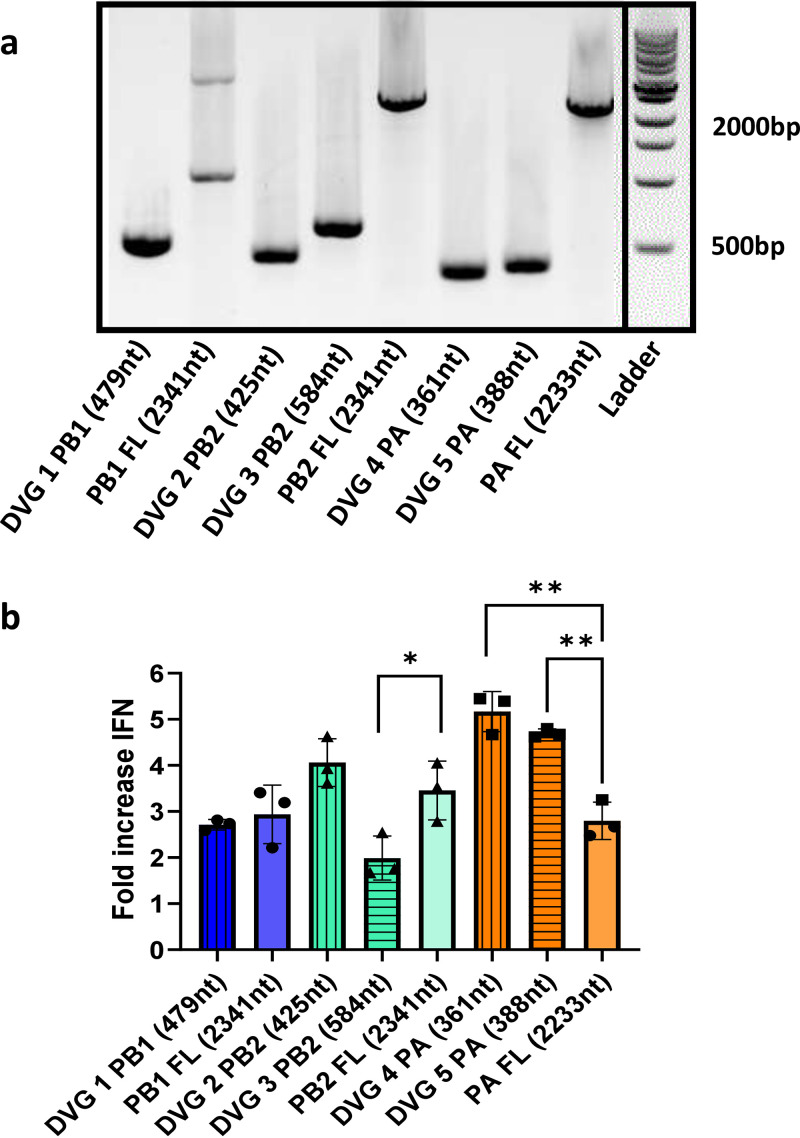
DVGs cloned from the 7:1 Tky/05 virus are immunostimulatory. (a) Gel electrophoresis showing RT-PCR products of full-length (FL) genome or DVGs using segment-specific terminal primers for the polymerase genes. RNA from HEK293T cells transfected with 3P (PB1, PB2, and PA) and NP expression plasmids, reporter plasmids, and DVG or FL genome template was extracted 24 h posttransfection. The gel was spliced to remove wells for clarity and labeling alignment with panel b. (b) IFN-β promoter activation induced by the replication of either full-length segment or DVG. Bars and error bars represent mean values ± SD (*n* = 3, representative of 3 independent experiments). Differences between each DVG and the full-length segment it was derived from were calculated by the two-sided unpaired *t* test. *, *P* < 0.05; **, *P* < 0.01.

To confirm that the DVGs were capable of inducing type I IFN expression, we cloned identified DVGs from the 7:1 Tky/05 LOW virus stock into plasmids and expressed the RNAs from the polymerase I promoter. We drove amplification of the DVGs or their corresponding full-length (FL) polymerase segments by cotransfecting plasmids for expression of the viral polymerase and NP into HEK293T cells and measured the IFN-β promoter activity induced by their replication. All of the DVGs and FL segments were successfully amplified (Fig. 8a), and all activated the IFN-β promoter (Fig. 8b). PA DVGs demonstrated the highest IFN-β activity. Interestingly, only these PA DVGs were more immunostimulatory than their cognate FL segment, while all of the other DVGs either resulted in a level of IFN-β promoter activity similar to the level seen with the FL segment or, in the case of PB2 DVG 3 (584 nt), significantly less activity. These results demonstrate that not all DVGs trigger type I IFN equally in a minigenome assay and suggest that the PA DVGs contribute to innate immune activation to a greater extent than those derived from the PB1 or PB2 segment.

## DISCUSSION

DVGs are no longer believed to be simply by-products of culturing RNA viruses at high MOIs *in vitro*; they have been detected in natural and experimental infections and even found to play a key role in modulating clinical outcomes (43, 44). DVGs act as potent triggers for cytokine responses that could either confer protection on the host (36, 45) or contribute to immunopathology (20). These different outcomes likely depend on the timing of virus replication and DVG generation and amplification and the consequent innate responses that can only be assessed in *in vivo* studies. To date, the majority of *in vivo* animal studies addressing the role of influenza DVGs have used well-characterized laboratory strains, such as WSN or PR8 (33, 36). However, infections with these strains may not adequately reflect the same outcome as HPAIV mammalian infections, which are more commonly associated with immunopathological responses (20, 46). Here, we utilized a virus containing the internal genes of an HPAIV H5N1 subtype and show that the amount of DVGs in the starting inoculum can have a profound impact on the pathogenesis in a BALB/c mouse model. We found that a stock with a high level of DVGs (7:1 Tky/05 HIGH) was attenuated in mice, resulting in minimal disease. In contrast, infection with a virus stock with a lower starting DVG content but otherwise equal genetic background (7:1 Tky/05 LOW) resulted in a severe outcome.

The observation that high levels of DVGs present in the initial inoculum attenuate influenza virus infection agrees with previous studies (29, 36, 45). The abundant DVGs in the 7:1 Tky/05 HIGH stock most likely reduce the amplification of infectious virus both by activating a robust early type I IFN response and by competing with the standard full genome for replication and for viral resources and packaging, due to their shorter size (34, 35, 47, 48). This is supported by our *in vitro* data, where the 7:1 Tky/05 HIGH virus replicated poorly in MDCK and A549 cells when infectivity was measured by PFU but triggered an earlier type I IFN response and release of proinflammatory cytokines. In the lungs of the 7:1 Tky/05 HIGH-infected mice, there was very early (6 to 24 h.p.i.) production of IFN-α and IL-1β, both of which have been associated with a protective host response (49, 50). This early suppression of viral replication that promotes survival of the host is a similar outcome to that seen in studies where DIPs were administered prior to influenza A virus (IAV) challenge (51).

When DVG levels were not as grossly elevated, a different phenotype emerged, where the two viruses passaged at a low MOI (7:1 Tky/05 LOW and 6:2 Tky/05) showed different outcomes in mice. There were differences in viral loads between the 6:2 Tky/05 and 7:1 Tky/05 LOW viruses, with modest increases in infectious virus (measured by PFU) at 24 and 48 h.p.i. in the murine lungs for the 7:1 Tky/05 LOW virus. However, there was no significant difference in growth kinetics in cell culture. Although high viral load can be associated with fatal human influenza infections, a severe outcome is more closely correlated with excessive cytokine production and immunopathology (52, 53). Previous work has shown that DVGs are potent ligands for RIG-I (38, 39), as well as other PRRs, such as ZBP1, which can drive necroptosis (54), and the production of cytokines coincides with DVG emergence following infection with influenza virus (36). Indeed, our initial mouse experiment supports this, as infection with the 7:1 Tky/05 stock caused more rapid weight loss than infection with the 6:2 Tky/05 stock, but smaller amounts of infectious virus were recovered from the lungs. In the latter mouse experiment, the 7:1 Tky/05 LOW virus had more abundant DVGs in the initial viral stock than the 6:2 Tky/05 virus and higher levels of DVGs at all time points in the murine lungs as determined by NGS; this disparity in DVGs could have contributed to this difference in immune activation. Indeed, at 24 h.p.i., higher numbers of inflammatory cells were recruited to the BAL fluid, and at 48 h.p.i., higher levels of proinflammatory cytokines and chemokines were induced by the 7:1 Tky/05 LOW virus than by the 6:2 Tky/05 virus. This high proinflammatory cytokine/chemokine signature is in keeping with other studies examining cytokine induction in severe influenza infections and reinforces that hypercytokinemia contributes to H5N1 pathogenesis (4, 55).

The timing of DVG generation and amplification likely contributes to the profound differences in severity. A recent study examining cbDVG accumulation in human nasal wash/nasopharyngeal swabs from respiratory syncytial virus (RSV)-infected individuals suggested that prolonged and later cbDVG production led to a more severe clinical outcome, whereas DVGs produced early during infection led to more mild disease (15). Analysis of the abundance of DVGs in the murine lungs showed that the 7:1 Tky/05 HIGH virus generated higher levels at early time points, whereas at the latest time point sampled (96 h.p.i.), DVG levels were highest from the 7:1 Tky/05 LOW virus-infected mice. We propose that in these mice, there were insufficient amounts of DVGs to trigger type I IFN and proinflammatory cytokines at the early stage of infection (6 to 24 h.p.i.), resulting in uncontrolled viral replication. However, by the time DVGs accumulated to trigger innate immunity (48 h.p.i.), the viral loads were already high and immunopathology occurred instead, analogous to exacerbated disease following treatment of mice with IFN-α when administered during active viral replication (56).

Why some DVGs are more immunostimulatory than their parental full-length genome is still not fully understood, considering they share the same termini (39). Although the immunostimulatory properties of IAV DVGs have been attributed to their smaller size compared to the full-length genome and their greater abundance, recent findings suggests that type I IFN induction by RNA PAMPs is length independent (34). It therefore could be that, as in *Paramyxoviridae* infections, some DVGs have an altered secondary structure, making them more visible to RIG-I (57), or fail to package properly into the viral RNP complex. Indeed, a recent preprint identifies t-loops formed by some influenza mvRNAs as disrupting RNA polymerase processivity and, thus, enabling RIG-I binding (58). Reduction of influenza virus nucleoprotein (NP) expression has also been shown to promote both DVG and mvRNA generation, leading to an enhanced host antiviral response (59). It has been proposed that DVGs may not be properly encapsidated, which would increase accessibility to RIG-I by leaving parts of the RNA exposed (60). Single-cell sequencing studies have shown that diverse DVG species generated throughout an influenza infection vary in their ability to stimulate an innate immune response (61). Indeed, our findings show that the PA DVGs are able to induce higher IFN expression than FL PA in a minigenome assay, but this is not observed with the PB1 or PB2 DVGs. It is therefore entirely feasible that different DVG species vary in their level of NP encapsidation and that this could influence host innate immune recognition.

Our studies highlight the need for care to be taken when generating virus stocks, especially if comparisons between different viruses are to be made regarding host immune responses. By using low MOIs for preparation of viral stocks and short incubation times, we tried to limit the propagation of DVGs and therefore reduce the number of noninfectious particles, as recommended by Xue et al. (62). However, preparation of completely DVG-free viral stocks is challenging. DVGs were generated by all rescued viruses, even after one passage directly following the original rescue, as seen in the 6:2 Tky/05 stock. The 7:1 Tky/05 LOW stock, generated when passaging the 7:1 Tky/05 virus at an MOI of 0.0001, still contained DVGs visible by RT-PCR; although this stock did have a significantly reduced genome copy number-to-PFU ratio compared with that of the original 7:1 Tky/05 stock (813:1 versus 40:1). This is consistent with other findings where DVG content could be reduced by passaging at a low MOI but was still detectable either by RT-PCR or NGS technologies (21, 41, 63). It is therefore essential that both infectious and noninfectious particles are considered when designing experiments comparing influenza strains. This could be achieved by calculating genome/PFU ratios on virus stocks and ensuring these are similar or by normalizing input by assays quantifying total particles, and not just infectious particles, as measured by PFU or 50% tissue culture infective dose (TCID_50_).

There are some limitations to our study. By propagating virus stocks at a high MOI, the amounts of noninfectious particles increased, and although some of these would be particles containing DVGs, other noninfectious particles would also be enriched. Semi-infectious particles, which lack all eight viral segments and therefore also require complementation to be fully infectious, are abundantly produced by IAVs and have been shown to have an impact on the virus population dynamics (64–66). Single-cell studies have revealed that either the absence of expression of the NS gene or the presence of a mutated NS1 protein is a significant contributor to the innate immune response, along with deletions and mutations in the PB1 gene (67). As our analysis only focused on DVGs, we cannot address the role that other viral defects have in modulating the outcome of infection. The mismatch in the NA segments between the 6:2 Tky/05 and 7:1 Tky/05 LOW viruses also makes it harder to fully assess the impact that the kinetics of DVGs had on the differences in pathogenicity observed between these two viral infections. The increased weight loss and morbidity caused by the 7:1 Tky/05 LOW virus could simply be attributed to its different NA segment, as the 7:1 Tky/05 LOW virus had the Tky/05 NA, whereas the 6:2 Tky/05 virus had the PR8 NA. However, it should be noted that we have observed a weight loss curve similar to that of the 7:1 Tky/05 LOW virus-infected mice presented in this study when using a different preparation of the 6:2 Tky/05 virus previously (32), suggesting that factors other than the NA segment contributed to this difference in disease severity.

Overall, we show that an increased level of noninfectious particles, including DVGs, influences the pathogenicity of a virus containing the internal genes from the HPAIV H5N1 subtype following infection in BALB/c mice. While the high-DVG virus (7:1 Tky/05 HIGH) was nonpathogenic, another stock (7:1 Tky/05 LOW), which induced the rapid accumulation of DVGs after infection, led to a higher viral lung load, higher levels of proinflammatory cytokines in the lung homogenates, and increased morbidity. This is in stark contrast to the 6:2 Tky/05 stock, which upon infection, only accumulated low levels of DVGs, where most mice survived and ultimately cleared the virus. The exact role individual DVGs have in influenza pathogenesis remains unresolved, but our research suggests that DVG generation may not always be beneficial for the host and that DVG kinetics impact the activation of host innate immune responses, viral replication, and ultimately, the infection outcome.

## MATERIALS AND METHODS

### Ethics statement.

All work was approved by the local genetic manipulation (GM) safety committee of Imperial College London, St Mary’s Campus (center number GM77), and the Health and Safety Executive of the United Kingdom and was carried out in accordance with the approved guidelines. All animal research described in this study was approved by the Animal Welfare and Ethical Review Board (AWERB) at Imperial College London and carried out under a United Kingdom Home Office license, no. P48DAD9B4, in accordance with the approved guidelines.

### Cell lines.

Madin-Darby canine kidney (MDCK; ATCC), adenocarcinomic human alveolar basal epithelial (A549; ATCC), and human embryonic kidney 293T (HEK293T; ATCC) cells were grown in Dulbecco’s modified Eagle’s medium (DMEM; Invitrogen) supplemented with 10% fetal bovine serum (FBS; LabTech), 1% penicillin-streptomycin (Invitrogen), and 1% nonessential amino acids (NEAA; Gibco). An A549 cell line containing a stable integrate of the firefly luciferase gene driven by the IFN-β promoter (A549 IFN-β Luc cells) was maintained as described above but was supplemented with 2 mg/mL geneticin (Gibco). All cells were grown at 37°C and 5% CO2.

### Generation and infection of mouse BMDMs.

To isolate bone marrow cells, the femurs and tibias of 6- to 8-week-old female BALB/c mice were excised and cleaned of flesh. Bone marrow cells were flushed out, filtered through a nylon cell strainer (Falcon), and washed with phosphate-buffered saline (PBS). Cells were resuspended and differentiated in RPMI 1640 medium (10% FBS, 0.05 mM β-mercaptoethanol, 1% l-glutamine, and 1% penicillin/streptomycin) supplemented with 20% L-929 cell supernatant. On day 3 of culture, nonadherent cells were removed and fresh medium containing the same concentration of L929 cell supernatant was added. On day 7, cells were harvested for further experimental use. Macrophages were seeded at 1.25 × 10^5^ cells per well and rested for 24 h before stimulation with influenza virus or medium controls, and at appropriate time points, supernatants were collected and cells washed once in PBS and lysed with TRIzol (Thermo Fisher Scientific) and frozen at −80°C.

### Virus.

The viruses used in this study were either 7:1 or 6:2 reassortant viruses with all internal segments from A/turkey/Turkey/1/2005 (H5N1). The 7:1 Tky/05, 7:1 Tky/05 HIGH, and 7:1 Tky/05 LOW virus stocks contained the HA from A/Puerto Rico/1934 (H1N1) and the NA from A/turkey/Turkey/1/2005. The 6:2 Tky/05 LOW virus contained both the HA and NA segments from A/Puerto Rico/8/1934. All virus stocks were originally rescued by reverse genetics and grown on MDCK cells at 37°C and 5% CO_2._ Briefly, 12 plasmids, comprising the 8 pPolI plasmids encoding the virus segments and 4 helper pCAGGS expression plasmids, were transfected into 293T cells and cocultured with MDCK cells. Serum-free DMEM supplemented with 1 μg/mL of TPCK L-(tosylamido-2-phenyl) ethyl chloromethyl ketone trypsin (Worthington-Biochemical) was used for infection, and viruses were harvested once CPE was observed. Viruses were further passaged once in MDCK cells at either an MOI of 0.0001 (6:2 Tky/05 and 7:1 Tky/05 LOW) or an MOI of 0.01 (7:1 Tky/05 HIGH) and harvested 48 h later. After harvesting, all viruses were clarified by centrifugation and stored at −80°C. Viral titers were determined by plaque assay.

### Viral growth curves.

Six-well plates of confluent MDCK or A549 cells were infected with viruses at an MOI of 0.001 (MDCK) or 0.01 (A549). Cells were maintained in serum-free medium with the addition of 1 μg/mL TPCK trypsin (Worthington-Biochemical). Supernatant samples were taken at indicated time points and frozen until viral quantification by plaque assay on MDCK cells.

### Infectivity/total particle ratio.

Infectivity/total particle ratios were determined as described by Xue et al. (62). Briefly, to quantify the amounts of infectious particles, plaque assays were performed and the numbers of PFU per 25 μL were calculated. Total particles were determined by hemagglutination assay using 0.7% turkey red blood cells in 25-μL volumes.

### Virus infections in A549 and A549 IFN-β Luc cells.

Triplicate wells of a 24-well plate of confluent A549 or A549 IFN-β Luc cells were infected with an MOI of 1 PFU/cell. After 1 h, virus inoculums were removed and replaced with 2% DMEM. For A549 cells, at specified time points, supernatants were harvested and cells washed in PBS before lysis in TRIzol. Both the supernatant and the cell lysate were stored at −80°C. Following infection in A549 IFN-β Luc cells, cells were washed once in PBS before the addition of passive lysis buffer (Promega) followed by one freeze/thaw cycle, and luciferase was measured using the luciferase assay system (Promega) with a FLUOstar Omega plate reader (BMG Labtech).

### RNA extraction from viruses.

Amounts of 250 μL from virus stocks were added to 750 μL TRIzol LS (Thermo Fisher Scientific), 200 μL chloroform was added, and centrifugation was performed at 10,000 × *g* at 4°C for 20 min. The aqueous phase was removed, equal volumes of 100% ethanol added and mixed, and the mixtures loaded onto Zymo RNA clean & concentrator 5 columns.

### RNA extraction from cells and murine lungs.

Total RNA was extracted by adding appropriate volumes of chloroform and centrifuging at 10,000 × *g* at 4°C for 20 min. The aqueous phase was then removed, equal volumes of 100% ethanol added and mixed, and the mixtures loaded onto either Zymo RNA clean & concentrator 5 columns (for macrophages) or Zymo RNA miniprep columns (for murine lung homogenates or A549 cells). On-column DNase I treatment was performed on all RNA samples.

### RT-qPCR for M gene detection.

RT-qPCR using AgPath-ID one-step RT-PCR reagents (Thermo Fisher) in 20-μL reaction mixtures was performed using the 7500 real-time PCR system (ABI). Five microliters of RNA was added to a mastermix containing 10 μL RT-PCR buffer (2×), 0.8 μM forward primer (5′-GACCRATCCTGTCACCTCTGA-3′), 0.8 μM reverse primer (5′-AGGGCATTYTGGACAAAKCGTCTA-3′), 0.4 μM probe (5′ FAM-TGCAGTCCTCGCTCACTGGGCACG-BHQ1-5′), and 1 μL RT-PCR enzyme mix (25×). The following conditions were used: 45°C for 10 min for 1 cycle; 95°C for 10 min for 1 cycle; 95°C for 15 s and then 60°C for 45 s for 40 cycles. For each sample, the threshold cycle (*C_T_*) value for the target M gene was determined. Based on the standard curves, absolute M gene copy numbers were calculated.

### RT-PCR for full-length genome and DVG detection.

Either 500 ng (A549 cells), 100 ng (BMDMs), or 2 μg (murine lung homogenates) of total RNA was used to make cDNA using the Uni12 primer (5′-AGCGAAAGCAGG-3′) and superscript IV (Life Technologies) according to the manufacturer’s instructions. For cDNA synthesis from viral stocks, 3 μL RNA was added to each RT reaction mixture and the reactions performed as described above. RT-PCR for the polymerase segments was used to determine the presence of DVGs and full genomes. To detect both DVGs and full genomes, the following terminal primers were used: PB1 Forward (5′-AGCGAAAGCAGGCAAACC-3′), PB1 Reverse (5′-AGTAGAAACAAGGCATTTTTTCACG-3′), PB2 Forward (5′-AGCGAAAGCAGGTCAAATATATTC-3′), PB2 Reverse (5′-AGTAGAAACAAGGTCGTTTTTAAAC-3′), PA Forward (5′-AGCGAAAGCAGGTACTGATTC-3′), and PA Reverse (5′-AGTAGAAACAAGGTACTTTTTTGG-3′). All PCRs were performed using 2 μL of cDNA in each reaction mixture, with KOD polymerase (Merck).

### TOPO cloning of DVGs.

PCR products ranging from 300 to 1,000 bp were gel purified and cloned into the pCR-blunt II TOPO vector (Thermo Fisher Scientific). The TOPO-ligated PCR products were transformed into NEB 5-alpha competent E. coli cells (New England BioLabs). Colonies were grown in Luria-Bertani medium, and plasmids were extracted using the alkaline lysis method (Monarch). The inserts were then Sanger sequenced.

### Subcloning of DVGs into pPolI plasmids.

PB1, PB2, and PA DVGs previously cloned into TOPO vectors from the 7:1 Tky/05 LOW viral stock were subcloned into the pPolI vector using segment-specific primers containing BsaI or BsmBI restriction sites (68), followed by BsaI/BsmBI digestion and subsequent ligation. Colonies were grown in Luria-Bertani medium, plasmids extracted using the alkaline lysis method, and inserts Sanger sequenced prior to generation of plasmid DNA using a HiSpeed Plasmid Maxi Kit (Qiagen).

### Minigenome-driven luciferase reporter IFN expression assays.

pCAGGs expression plasmids encoding PB1 (0.1 μg), PB2 (0.1 μg), and PA (0.05 μg) (3P) and NP (0.2 μg) from Tky/05 were transfected into HEK293T cells alongside 0.1 μg pPolI plasmids containing either a Tky/05 polymerase full-length segment or a polymerase DVG. Additionally, 0.1 μg of a firefly luciferase reporter plasmid under the control of the *IFNB* promoter and 0.025 μg of a pCAGGs expression plasmid encoding *Renilla* luciferase were cotransfected using Lipofectamine 3000 (Thermo Fisher). Transfections were also performed where the PB2 pCAGGs plasmid was omitted (2P) and empty pCAGGs transfected instead. Cells were lysed 24 h posttransfection in passive lysis buffer (Promega), and luciferase was measured using the dual-luciferase assay system (Promega) with a FLUOstar Omega plate reader (BMG Labtech). Values were normalized to the value for *Renilla* luciferase and expressed as the fold increase over the value for 2P. Minigenome assays were also performed to confirm amplification of the DVG or full-length genome by RT-PCR as described above but using a tagged Uni12 primer (5′-GGCCGTCATCGGCCATTAGCRAAAGCAGG-3′; the tag is underlined) for cDNA synthesis and tagged forward primer (5′-GGCCGTCATCGGCCATT-3′) with relevant terminal primers in the PCR mixture to ensure no residual DNA from the plasmid was detected.

### Chemokine and cytokine detection.

The quantities of the cytokines IFN-γ, IL-6, TNF, IP-10, MCP-1, and MIP-1β were determined by the meso scale discovery service as a 10-spot U-PLEX kit (catalog number K15069L-2) in 100 μL of BAL fluid. The concentrations of IFN-α, TNF, and IL-1β in mouse lung tissue were measured with the VeriKine mouse IFN-α ELISA kit (catalog number 42120; PBL), Quantikine mouse TNF ELISA kit (catalog number MTA00B; R&D Systems), and Quantikine mouse IL-1β/IL-1F2 kit (catalog number MLB00C; R&D Systems). The concentrations of IFN-β in A549 cell supernatants were measured with the VeriKine human IFN-β ELISA kit (catalog number 42400; PBL).

For qPCR detection of cytokines, cDNA was synthesized by using a high-capacity cDNA reverse transcription kit (Applied Biosystems) using either 500 ng total RNA (A549 cells) or 200 ng total RNA (BMDMs). The mRNA levels of *Tnf* and *Il6* in macrophages and *IFNB* in A549 cells were tested with SYBR green PCR mix (Applied Biosystems). The primers used were as follows: murine *Tnf* forward (5’GGCAGGTCTACTTTGGAGTCATTG-3′), murine *Tnf* reverse (5′-ACATTCGAGGCTCCAGTGAATTCGG-3′), murine *Il6* forward (5′-GACAAAGCCAGAGTCCTTCAGAGAG-3′), murine *Il6* reverse (5′-CTAGGTTTGCCGAGTAGATCTC-3′), human *IFNB* forward (5′-GCCGCATTGACCATCT-3′), and human *IFNB* reverse (5′-CACAGTGACTGTTACTCCT-3′). To detect murine *Ifna5* mRNA, a TaqMan gene expression assay (assay identification number [ID] Mm00833976_s1; Thermo Fisher Scientific) was used alongside a glyceraldehyde-3-phosphate dehydrogenase (*Gapdh*) gene expression assay (assay ID Mm99999915_g1; Thermo Fisher Scientific). qPCR analysis was carried out in duplicate on a ViiA 7 real-time PCR system (Thermo Fisher). Fold changes in gene expression relative to the expression in mock-infected controls were calculated using the 2^−ΔΔ^*^CT^* method, with GAPDH expression as an internal control.

### Mouse experiments.

Six- to 8-week-old female BALB/c mice (Envigo RMS UK Ltd.) were maintained in pathogen-free conditions until used for viral infection. Mice were anesthetized using isoflurane and infected intranasally with 10^5^ PFU influenza virus in a 25-35-μL volume or treated with sterile PBS (mock infection). Animals were monitored and weighed daily. Lungs were harvested at designated time points or when weight loss dropped below 80% of the original weight on day zero. Lungs were split into three equal parts, weighed, suspended in 1 mL PBS (for plaque assays), 1 mL TRIzol (RNA extraction), or 350 μL protease inhibition buffer (1 tablet in 10 mL PBS; Roche) (for ELISA), and homogenized using 2.8-mm beads. The lungs homogenized in protease inhibition buffer were spun for 10 min at 10,000 × *g* at 4°C, and supernatants were transferred into fresh reaction tubes containing 650 μL protease inhibitor buffer. All were stored at −80°C. BAL fluid samples were collected by instilling the lungs with PBS. Supernatants were collected after centrifugation and assayed.

### NGS from murine lungs.

RNA from two murine lungs for each group (7:1Tky/05, 7:1Tky/05 HIGH, 7:1Tky/05 LOW, and 6:2 Tky/05 LOW infections) per time point (6, 24, 48, and 96 h.p.i.) were selected for NGS analysis. Amounts of 2 μg total RNA were used in cDNA reaction mixtures to amplify all eight genome segments using 2 μM MBTUni-12 primer (5′-ACGCGTGATCAGCRAAAGCAGG-3′) and SuperScript IV (Invitrogen). For PCR amplification, 10 μM MBTUni-12 (as above) and 10 μM MBTUni-13 primer (5′-ACGCGTGATCAGTAGAAACAAGG-3′) were incubated with 2 μL cDNA, and KOD polymerase was used (Merck). The PCR conditions were as follows: 95°C for 2 min, followed by 25 cycles of 95°C for 20 s, 57°C for 10 s, 70°C for 50 s, and a terminal extension of 72°C for 5 min. All samples were purified using the Monarch PCR cleanup kit (NEB). PCR products were diluted to 20 ng in 50 μL and fragmented using the Covaris M220 ultrafocused sonicator (Covaris). Libraries were constructed using the NEBNext Ultra II DNA library prep kit (NEB). Adapters were diluted 1:10 with 10 nM Tris/NaCl, and after ligation, samples were cleaned up using AMPure XP beads (Beckman-Coulter) without size selection. For PCR enrichment, index and universal primers were added using NEBNext multiplex oligonucleotides for Illumina (NEB) and 6 PCR cycles performed. A further cleanup step was performed using the AMPure XP beads, and libraries were quantified by Qubit (Thermo Fisher Scientific) and pooled at an equimolar ratio. Libraries were sequenced with paired-end 2 × 150-nt reads on an Illumina MiSeq using V2 chemistry. Fastq files were generated and demultiplexed with the bclfastq version 2.20 conversion software (Illumina). One set of sequencing reads from each pair (R1) were analyzed by ViReMa (version 0.10) (68) to detect junction spanning reads (DVGs). A read support cutoff (RSC) of >30 was used to minimize the number of inaccurate junctions.

### Statistical analysis.

Statistical analyses throughout this study were performed using GraphPad Prism version 9.0 (GraphPad Software) and are described in the figure legends.

### Data availability.

Raw sequences for mouse lung NGS data were deposited at http://www.ebi.ac.uk/ena, project number PRJEB56225.
