# Supplementary material for: Levels of Influenza A Virus Defective Viral Genomes Determine Pathogenesis in the BALB/c Mouse Model
Source: J Virol. 2022 Oct 13;96(21):e01178-22. doi: 10.1128/jvi.01178-22 (PMC9645217; doi:10.1128/jvi.01178-22)
Supplement: Supplemental file 1 — Table S1. Download jvi.01178-22-s0001.pdf, PDF file, 0.1 MB [file jvi.01178-22-s0001.pdf]

Supplementary Table 1. Summary of NGS data compiled from murine lung following ViReMa analysis.
Total viral reads are shown for each sample and DVGs with the deletion junctions shown and number of reads in parenthesis.
Those in bold and underlined have the same deletion junctions as those identified in the corresponding viral stock (only applies to DVGs identified from polymerase segments).

Mouse	Virus	Time Point (hours)	Total viral reads	PB1 DVGs	PB2 DVGs	PA DVGs	NP DVGs	HA DVGs
1	6:2 Tky/05	6	16,173	<30	<30	<30		
2	6:2 Tky/05	6	17,291	<30	**277-1872 (39)**	<30		
1	6:2 Tky/05	24	59,623	<30	**277-1872 (45)**	<30		
2	6:2 Tky/05	24	99,375	<30	**277-1872 (44), 209-1927 (42)**	<30	243-919 (110)	
2	6:2 Tky/05	48	155,424	<30	**209-1927 (312), 277-1872 (245),** 162-2138 (56)	295-1812 (30)		
2	6:2 Tky/05	96	157,018	<30	120-2138 (190), 162-2097 (67), **277-1872 (67),** 165-2138 (66), 190-2039 (52), 129-2140 (45), 217-2031 (44)	138-1958 (46)	243-919 (696)	
1	7:1 Tky/05 HIGH	6	88,686	**244-2107 (1083)**	**116-2033 (6076) 242-2000 (696)**, 149-2068 (331), 158-2114 (68), 110-2130 (53)	129-2002 (105), 128-1974 (55), 162-1971 (36)		
2	7:1 Tky/05 HIGH	6	72,724	**244-2107 (1074),** 210-2308 (54), 361-2040 (32)	**116-2033 (5138), 242-2000 (601)**, 149-2068 (339), 158-2114 (133), 110-2130 (51), 243-2121 (33)	**129-2002 (91), 128-1974 (77)**		91-1510 (37)
1	7:1 Tky/05 HIGH	24	38,529	244-2107 (425)	**116-2033 (2625), 242-2000 (441)**, 149-2068 (195), 158-2114 (48), 110-2130 (38)	**129-2002 (31)**		
2	7:1 Tky/05 HIGH	24	65,308	**244-2107 (431)**	**116-2033 (3597), 242-2000 (517)**, 149-2068 (259), 158-2114 (79)	**128-1974 (40)**	243-919 (158)	
1	7:1 Tky/05 HIGH	48	132,566	**244-2107 (1074),** 210-2038 (54), 361-2040 (32)	**116-2033 (14659), 242-2000 (1574)**, 149-2068 (1018), 158-2114 (285), 110-2130 (66), 244-2027 (64), 243-2121 (51), 215-2003 (35)	**128-1974 (155), 129-2002 (81), 187-1971 (38)**		
2	7:1 Tky/05 HIGH	48	56,589	**244-2107 (449)**	**116-2033 (6575), 149-2068 (506),** 242-2000 (140), 158-2114 (102),110-2130 (38)	**129-2002 (64), 128-1974 (58)**		
1	7:1 Tky/05 HIGH	96	351,152	**244-2107 (1727)**	**116-2033 (12135), 242-2000 (1759),** 149-2068 (648), 190-2039 (317), 158-2114 (94), 110-2130 (90), 243-2121 (83), 215-2019 (46), 215-2003 (34)	**129-2002 (85), 128-1974 (33)**	243-919 (1128)	
2	7:1 Tky/05 HIGH	96	215,109	**244-2107 (672)**	**116-2033 (8542), 242-2000 (1621)**, 149-2068 (620), 158-2114 (277), 110-2130 (62), 243-2121 (35)	1**29-2002 (148), 128-1974 (41), 187-1971 (39)**	243-919 (640)	
1	7:1 Tky/05 LOW	6	28,532	**244-2107 (190)**	**116-2033 (679), 242-2000 (65)**	**129-2002 (31)**	243-919 (141)	
2	7:1 Tky/05 LOW	6	45,852	**244-2107 (283)**	**116-2033 (1087), 242-2000 (155),** 149-2068 (39)	**128-1974 (70), 129-2002 (63)**		
1	7:1 Tky/05 LOW	24	243,849	**244-2107 (1380),** 231-2097 (36), 210-2038 (31)	**116-2033 (5844), 242-2000 (594),** 149-2068 (239), 158-2114 (83), 110-2130 (49), 120-2118 (32)	**129-2002 (359), 128-1974 (298), 162-1971 (41)187-1971 (39)**	243-919 (1391)	
2	7:1 Tky/05 LOW	24	204,938	**244-2107 (1317),** 231-2097 (35)	**116-2003 (5049), 242-2000 (629),** 149-2068 (237), 175-2099 (62), 158-2114 (55), 110-2130 (48)	**129-2002 (318), 128-1974 (270), 187-1971 (72) 162-1971 (34)**		
1	7:1 Tky/05 LOW	48	143,696	**244-2107 (1895),** 210-2038 (33)	**116-2033 (9375), 149-2068 (535),** 242-2000 (444), 158-2114 (91),243-2121 (69), 175-2099 (68), 110-2130 (40)	**129-2002 (742), 128-1974 (605), 187-1971 (99), 162-1971 (59)**		
2	7:1 Tky/05 LOW	48	64,999	**244-2107 (1190),** 231-2097 (36)	**116-2033 (4613), 242-2000 (192),** 149-2068 (167), 158-2114 (141), 110-2130 (43)	**129-2002 (465), 128-1974 (344), 187-1971 (64)**		
1	7:1 Tky/05 LOW	96	161,646	**244-2107 (2147)**	**116-2033 (12176), 149-2068 (633),** 158-2114(335), 120-2118 (164), 242-2000 (148), 110-2130 (86), 169-2121 (37), 155-2067 (35), 119-2147 (30)	**129-2002 (1583), 128-1974 (1218), 162-1971 (204), 187-1971 (173),** 134-1973 (45)		
2	7:1 Tky/05 LOW	96	347,736	**244-2107 (4608),** 210-2308 (71), 231-2097 (57), 190-2092 (54)	**116-2033 (16880), 242-2000 (1922)**, 149-2068 (835), 158-2114 (789), 175-2046 (232), 110-2130 (165), 243-2121 (81), 120-2118 (64), 120-2138 (59), 175-2099 (36)	**128-1974 (2217), 129-2002 (1674),** 134-1973 (256), **187-1971 (207), 162-1971 (198),** 204-1973 (41), 164-1885 (32)		
